# Risk of infection in patients with early inflammatory arthritis: results from a large UK prospective observational cohort study

**DOI:** 10.1093/rheumatology/keaf312

**Published:** 2025-06-05

**Authors:** Maryam A Adas, Katie Bechman, Mark D Russell, Victoria Allen, Samir Patel, Mark Gibson, Ioasaf Karafotias, Kathryn Biddle, Benjamin Zuckerman, Kaiyang Song, Deepak Nagra, Edward Alveyn, Suma Mahendrakar, Meryem Nursoy, Fabiola Atzeni, Sarah Gallagher, Elizabeth Price, Mark Garton, Andrew Rutherford, Andrew P Cope, Sam Norton, James B Galloway

**Affiliations:** Centre for Rheumatic Disease, King’s College London, London, UK; Centre for Rheumatic Disease, King’s College London, London, UK; Centre for Rheumatic Disease, King’s College London, London, UK; Centre for Rheumatic Disease, King’s College London, London, UK; Centre for Rheumatic Disease, King’s College London, London, UK; Centre for Rheumatic Disease, King’s College London, London, UK; Centre for Rheumatic Disease, King’s College London, London, UK; Centre for Rheumatic Disease, King’s College London, London, UK; Centre for Rheumatic Disease, King’s College London, London, UK; Centre for Rheumatic Disease, King’s College London, London, UK; Centre for Rheumatic Disease, King’s College London, London, UK; Centre for Rheumatic Disease, King’s College London, London, UK; Centre for Rheumatic Disease, King’s College London, London, UK; Centre for Rheumatic Disease, King’s College London, London, UK; Centre for Rheumatic Disease, King’s College London, London, UK; Rheumatology Unit, Department of Internal and Experimental Medicine, University of Messina, Messina, Italy; NEIAA, British Society for Rheumatology, London, UK; NEIAA, British Society for Rheumatology, London, UK; Rheumatology Department, The Shrewsbury and Telford Hospital NHS Trust, Shropshire, UK; Rheumatology Department, King’s College Hospital, London, UK; Centre for Rheumatic Disease, King’s College London, London, UK; Centre for Rheumatic Disease, King’s College London, London, UK; Psychology Department, Institute for Psychiatry, Psychology and Neuroscience, King’s College London, London, UK; Centre for Rheumatic Disease, King’s College London, London, UK

**Keywords:** early rheumatoid arthritis, rheumatoid arthritis, infection, mortality, serious infections, initial treatment strategy, csDMARDs, methotrexate, corticosteroids

## Abstract

**Objective:**

To identify risk of serious infections (SI) according to initial conventional synthetic DMARDs (csDMARD) and CS, in patients recruited to the National Early Inflammatory Arthritis Audit.

**Methods:**

An observational cohort study was used, including adults in England and Wales with new diagnoses of RA between 2018 and 2023. The main outcome was SI events, defined as infections requiring hospitalization/or resulting in death. Secondary analyses evaluated SI-related mortality alone. Hazard ratios (HR) were calculated using cox proportional hazards models. Primary predictor was initial treatment strategy, with confounder adjustments.

**Results:**

A total of 17 472 patients were included, of whom 10 997 were on MTX-based strategies, 4540 on other csDMARDs and 13 680 received CS. There were 1307 SI events, corresponding to incidence rates (IR) per 100 person-years of 3.02 (95% CI 2.86–3.19) and 311 cases of SI-related mortality (IR 0.69, 95% CI 0.61–0.77). MTX-based strategies were associated with reduced risk of SI events compared with other csDMARDs (adjusted HR 0.72, 95% CI 0.63–0.82). In unadjusted models, CS was associated with higher risk of SI events, but in adjusted models this association was no longer significant (adjusted HR 0.99, 95% CI 0.87–1.12). Increasing age, being a current/or ex-smoker (relative to non-smoker), having a comorbidity, being seropositive and having high DAS based on 28 joint count (DAS28) were all associated with increased incidence of SI. One unit increase in baseline DAS28 increases the risk of SI event by 10%.

**Conclusion:**

MTX-based regimens associated with a reduced risk of SI compared with other strategies. Patient-level and disease-related factors at diagnosis are important predictors of SI in individuals with new RA.

Rheumatology key messages

- Evidence on serious infection risk in early RA remain limited.
- In early RA, MTX-based strategies were associated with low infection rates.
- Patient factors and disease severity, over drug choice, are the most relevant factors when assessing the infection risk in early RA.

## Introduction

Infections are among the common causes of morbidity and mortality in RA, driven by both immune dysfunction and immunosuppressive therapies [1, 2]. Understanding the interaction between RA treatment and infection risk is essential for optimizing patient outcomes [3, 4].

Over the past three decades, there has been a substantial shift in RA treatment strategies, with an emphasis on early intervention to optimize disease outcomes during the critical ‘window of opportunity’ [5]. Despite therapeutic advancements, CS are widely used in RA, while safety concerns remain debated [6]. Conventional synthetic DMARDs (csDMARDs) continue to form the cornerstone of RA maintenance therapy [7–9] with little evidence available that specifically considers the safety of treatments for early RA, as most available evidence comes from individuals with well-established disease and a high comorbidity burden.

Using the National Early Inflammatory Arthritis Audit (NEIAA) data we aimed to: (i) describe serious infection (SI) incidence in early RA; and (ii) compare SI risk across treatment strategies, including MTX and CS at diagnosis. A secondary aim was to evaluate SI-related mortality separately.

## Methodology

### Data source

NEIAA is a quality improvement initiative commissioned by the Health Quality Improvement Partnership, aimed at enhancing the quality of care for patients with early inflammatory arthritis. This is achieved by assessing healthcare services against six predefined metrics based on the National Institute for Health and Care Excellence guidelines (NICE) [10]. Full details on NEIAA collection have been previously published [11]. NEIAA collects data on adults referred to secondary care rheumatology services with suspected inflammatory arthritis. Patients with a confirmed inflammatory arthritis diagnosis are eligible for further follow-up.

Outcome data are obtained through linkage with the Office for National Statistics, Hospital Episode Statistics database in England and the Patient Episode Database for Wales, of which outcomes are recorded using the International Classification of Diseases, 10th Revision (ICD-10) codes. NEIAA is supported by patients’ groups, who contribute to study designs, interpretation and findings dissemination.

### Study population

We included adult (aged ≥18 years) patients in England and Wales with a clinician confirmed RA diagnosis enrolled in NEIAA from May 2018 to April 2023 [RA diagnosis was based on clinician assessment and although classification criteria (i.e. ACR/EULAR) are likely to have informed clinical decisions, this was not mandated or explicitly recorded in the audit dataset]. The data flow chart can be seen in Supplementary Fig. S1, available at *Rheumatology* online.

### Outcomes

The primary outcome of this study was the occurrence of a serious infection (SI) episode, defined as either a hospital admission due to infection or an infection-related death, where an infectious event was listed as the primary cause of the admission. A hospitalized infection was defined as an unplanned hospital admission within a government-funded healthcare system (the National Health Service in the UK), where hospitalization is reserved for patients with clinical compromise requiring inpatient management. We excluded new infectious complications occurring during hospital admissions for other primary causes. The secondary outcome was an infection-related mortality. Bacterial, viral, fungal and all other infectious related diseases were identified using a predefined ICD-10 code list (Supplementary Table S1, available at *Rheumatology* online) using the World Health Organization sheet for ICD-10 [12]. ICD codes were identified by two clinical researchers (M.A.A., I.K.). Disagreements were resolved after discussion with a third and fourth clinical researcher with expertise in infectious disease (V.A., J.B.G.).

Individuals were considered at risk from the date of their RA diagnosis until the date of the outcome of interest, death from another cause or 1 April 2023, whichever came first.

### Covariates

Patient demographics were tabulated at baseline, including age, gender, socioeconomic position [measured using the Index of Multiple Deprivation (IMD)] and smoking status (current smoker, ex-smoker and never smoked). Disease characteristics recorded included symptom duration prior to diagnosis, seropositivity status for RF and anti-CCP antibody, tender joint count (0–28 joints), swollen joint count (0–28 joints), patient-reported global assessment score (0–100 scale), CRP (mg/l) and/or ESR (mm/h). The DAS based on 28 joint count (DAS28) was calculated using either CRP or ESR, depending upon data availability. In our cohort, baseline DAS28 was predominantly based on CRP (94%), while only 6% of scores were derived using ESR. Data on comorbidities at baseline, included the presence of pre-existing diabetes, hypertension and chronic lung disease.

### Treatment strategies

Information on the initial treatment strategies (i.e. csDMARDs monotherapy, csDMARDs combination therapy and concomitant steroids) were collected by the treating clinician at baseline and 3 months (steroids use was collected at baseline). MTX-based strategies (monotherapy or in combination with other csDMARDs) and other csDMARDs strategies were defined based on information recorded in NEIAA at baseline and at 3 months (i.e. an individual was defined as being on a MTX strategy if this had been recorded by 3 months). Steroids use was considered as a separate adjuvant therapy at baseline—in NEIAA, steroid use is only captured at baseline, not at later time points. Treatment route of administration and doses are not recorded in NEIAA. A sensitivity analysis was undertaken to examine the SI risk (primary outcome) across individual csDMARDs monotherapies compared with MTX monotherapy.

### Statistical analysis

For continuous measures, data were described as medians and interquartile ranges (IQR). For categorical measures, absolute numbers and percentages were used. Treatment strategies were used as binary variables, as dose information was not collected. Inferential statistics (i.e. *P*-values) were not reported for differences in baseline characteristics due to the large sample sizes, and to avoid drawing inferences based upon multiple hypothesis tests. Fine and Gray proportional hazards survival models were used to identify factors associated with increased risk of SI occurring, accounting for death due to any non-infective cause as a competing risk. Single failure models were used for all analyses. Robust standard errors were estimated to account for clustering of patients within the health trusts. All models were adjusted for age, gender, socioeconomic position, smoking status, comorbidities, seropositivity status and baseline disease severity. Analyses adjusted for only age and gender are available in the Supplementary Materials, available at *Rheumatology* online.

Multi-level imputation model was used to reduce bias attributable to missing data in baseline covariates including comorbidities, social deprivation, baseline DAS28 and seropositivity. Full methodology for the imputation model is in the Supplementary File, available at *Rheumatology* online. Risks were reported as hazard ratios (HRs) and 95% CI. Graphical display of the event rates over time are presented using cumulative incidence plots.

For all models, statistical significance was assessed at the 5% level. No corrections for multiple hypothesis testing were made.

To compare the mortality event rates in NEIAA to the general population, age-band stratified standardized mortality ratios (SMR) were calculated using publicly available mortality data from England as the reference [13]. Infection-related mortality was published until 2022, therefore an average infection-related death for the general population was calculated from 2018 to 2022. A sensitivity analyses was performed excluding coronavirus disease 2019 (COVID-19)-related mortality. All statistical analyses were performed using Stata version 17.0 (StataCorp, College Station, TX, USA).

### Ethical approval

Approval to use the NEIAA dataset was obtained from Healthcare Quality Improvement Partnership (HQIP). Informed patient consent was not required, as NEIAA has permission from the UK Government Secretary of State for Health to collect data for the purposes of national audit. Ethical approval to undertake research in NEIAA has been granted (Clinical Advisory Group Reference: 19/CAG/0059; Research Ethics Committee reference: 19/EE/0082). Data access requests are made through HQIP and are subject to data sharing agreement approval.

## Results

### Population characteristics

A total of 17 803 patients with a confirmed diagnosis of RA were recruited to NEIAA between May 2018 and April 2023. Of those, 17 472 patients had data available on their initial treatment strategy using csDMARDs and were included in further analyses, with a mean follow-up time of 2.62 years (s.d. 1.52).

Full baseline characteristics of the included patients can be seen in Table 1. There was a predominance of females (*n* = 11 081; 63%), a mean age of 59 years (s.d. 15); diabetes mellitus (*n* = 1660; 10%), chronic lung disease (*n* = 1958; 11%) and hypertension (*n* = 3665; 21%). Some 12 070 (69.1%) of the cohort were seropositive for either RF or CCP antibody. Mean baseline DAS28 was 4.9 (s.d. 1.4). In total, 79% of the cohort (13 680/17 472) received concomitant CS as part of their initial treatment regimen. Details of the patient characteristics separated by steroid strategy are presented in the Supplementary Table S2, available at *Rheumatology* online. There were no missing data for age, gender or smoking status. Full details on missing data are shown in Supplementary Table S3, available at *Rheumatology* online.

**Table 1.** Baseline characteristics of patients with RA in NEIAA by treatment strategy

	Total patients starting csDMARD by three months	Any MTX strategy (mono or combination)	Any other csDMARD strategy	No DMARD^a^
Variables	*N* = 17 472	*N* = 10 997	*N* = 4540	*N* = 1935
Age, years, mean (s.d.)	59 (15)	59 (15)	59 (17)	60 (16)
Age band, *n* (%)				
16– years	324 (1.9)	197 (1.8)	94 (2.1)	33 (1.7)
25– years	1841 (10.5)	1004 (9.1)	621 (13.7)	216 (11.2)
40– years	8164 (46.7)	5330 (48.5)	1950 (43.0)	884 (45.7)
65– years	4053 (23.2)	2661 (24.2)	990 (21.8)	402 (20.8)
75– years	3090 (17.7)	1805 (16.4)	885 (19.5)	400 (20.7)
Gender, *n* (%)				
Male	6391 (36.6)	4135 (37.6)	1530 (33.7)	726 (37.5)
Female	11 081 (63.4)	6862 (62.4)	3010 (66.3)	1209 (62.5)
Smoking status, *n* (%)				
Current smoker	3327 (19.0)	2127 (19.3)	822 (18.1)	378 (19.5)
Ex-smoker	5043 (28.9)	3178 (28.9)	1345 (29.6)	520 (26.9)
Never smoked	7477 (42.8)	4690 (42.6)	1918 (42.2)	869 (44.9)
Not known	1625 (9.3)	1002 (9.1)	455 (10.0)	168 (8.7)
Social deprivation (IMD quintiles), *n* (%)				
1 (most deprived)	2889 (17.5)	1837 (17.7)	722 (16.8)	330 (17.6)
2	3427 (20.7)	2066 (19.9)	985 (22.9)	376 (20.0)
3	3492 (21.1)	2248 (21.7)	872 (20.3)	372 (19.8)
4	3258 (19.7)	2109 (20.3)	750 (17.4)	399 (21.3)
5 (least deprived)	3487 (21.1)	2119 (20.4)	969 (22.5)	399 (21.3)
Ethnicity, *n* (%)				
White	14 821 (84.8)	9509 (86.5)	3696 (81.4)	1616 (83.5)
Black	462 (2.6)	252 (2.3)	144 (3.2)	66 (3.4)
Asian	1371 (7.8)	744 (6.8)	473 (10.4)	154 (8.0)
Mixed	86 (0.5)	40 (0.4)	37 (0.8)	9 (0.5)
Other	552 (3.2)	340 (3.1)	147 (3.2)	65 (3.4)
Not known	180 (1.0)	112 (1.0)	43 (0.9)	25 (1.3)
Comorbidities, *n* (%)				
Diabetes mellitus	1660 (10)	1038 (9)	428 (9)	194 (10)
Hypertension	3665 (21)	2310 (21)	963 (21)	392 (20)
Lung disease	1985 (11)	1000 (9)	754 (17)	231 (12)
Serostatus, *n* (%)				
Seronegative	5402 (30.9)	3277 (29.8)	1428 (31.5)	697 (36.0)
RF positive	2676 (15.3)	1532 (13.9)	749 (16.5)	395 (20.4)
CCP positive	1850 (10.6)	1155 (10.5)	523 (11.5)	172 (8.9)
Double positive (RF and CCP)	7544 (43.2)	5033 (45.8)	1840 (40.5)	671 (34.7)
Duration of symptoms, *n* (%)				
<1 month	1380 (8.0)	873 (8.0)	361 (8.0)	146 (7.6)
1–3 months	5905 (34.0)	3811 (34.9)	1496 (33.1)	598 (31.2)
3–6 months	4171 (24.0)	2675 (24.5)	1043 (23.1)	453 (23.6)
6–12 months	3212 (18.5)	2021 (18.5)	837 (18.5)	354 (18.5)
>12 months	2682 (15.3)	1539 (13.9)	778 (17.1)	365 (18.8)
Baseline DAS28, mean (s.d.)	4.9 (1.4)	5.1 (1.4)	4.6 (1.5)	4.5 (1.6)
ESR mm/h, median (IQR)	27.0 (12.0, 45.0)	28.0 (13.0, 46.0)	26.0 (11.0, 44.0)	24.0 (9.0, 43.0)
CRP mg/L, median (IQR)	11.0 (4.0, 29.0)	13.0 (5.0, 32.0)	10.0 (4.0, 26.0	9.0 (4.0, 24.0)
Adjuvant baseline CS, *n* (%)	13 680 (79)	9141 (83)	3292 (73)	1247 (65)

a No DMARD strategy is when the patient was not started/entered a treatment strategy during the rheumatology visit by 3 months. NEIAA: National Early Inflammatory Arthritis Audit; csDMARD: conventional synthetic DMARD; DAS28: DAS based on 28 joint counts; IQR: interquartile range; IMD: Index of Multiple Deprivation.

MTX was the most used csDMARD strategy, with 10 997/17 472 patients (63%) receiving a MTX-based regimen either as monotherapy or in combination with another csDMARD. Overall, 4540/17 472 patients (26%) received a csDMARD regimen that did not include MTX and 1935/17 472 (11%) patients had delayed initiation of a csDMARDs by 3 months. A slightly higher proportion of patients who were not started on csDMARDs were over 75 years of age (20.7%). Patients not started on a csDMARD had lower baseline DAS28 [DAS28: 4.6 (s.d. 1.6)] compared with MTX group [DAS28: 5.1 (s.d. 1.4)] and other csDMARDs group [DAS28: 4.6 (s.d. 1.5)]. Numerically lower CRP and ESR levels, and a lower prevalence of seropositivity were seen in patients who did not start csDMARD. Comorbidity differences were also observed, with a higher proportion of lung disease in those not started on MTX-based strategies 754 (17%) compared with those on MTX 1000 (9%).

### SI events

**Figure 1.**
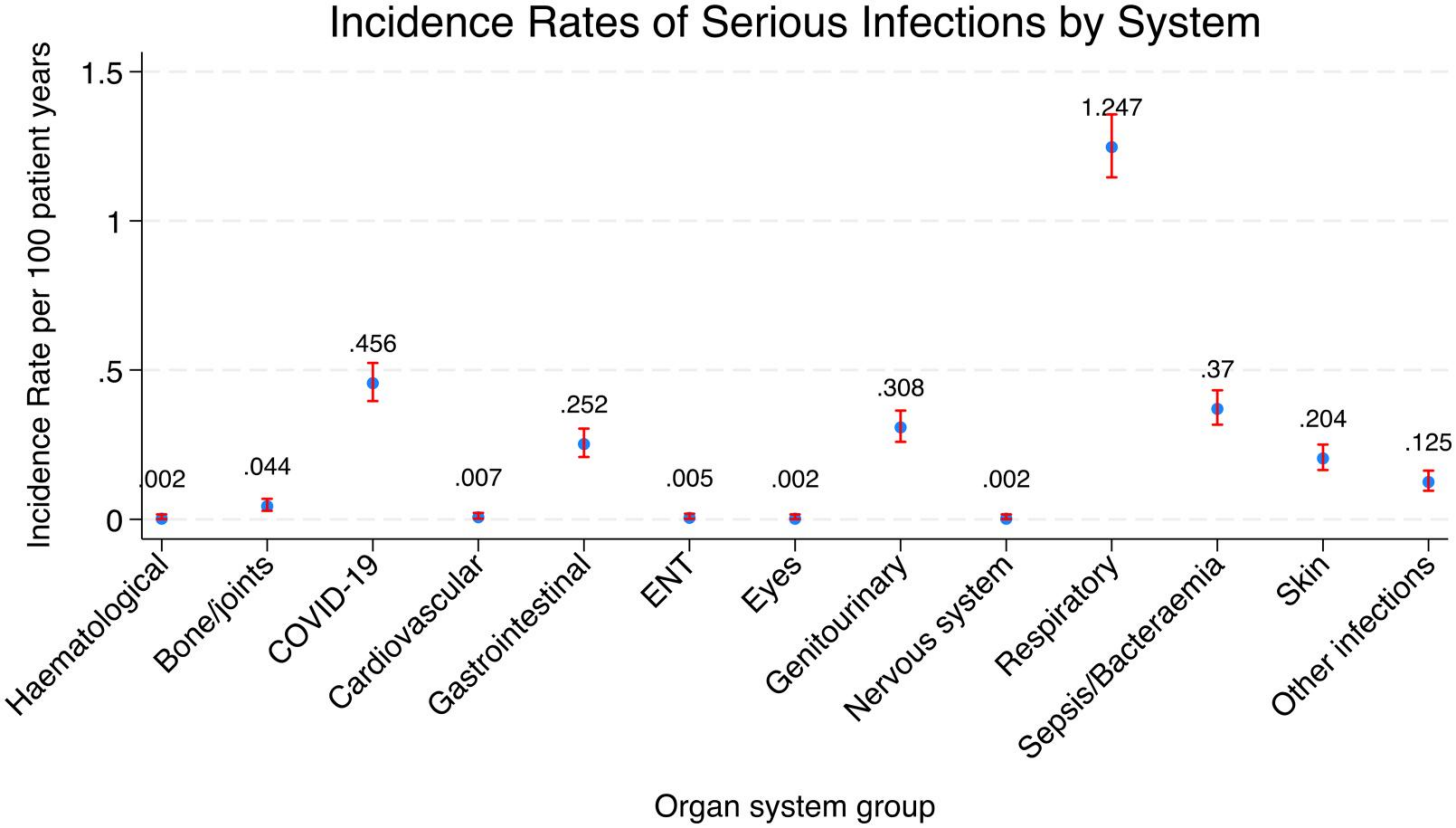
Incidence rates of serious infections in rheumatoid arthritis, stratified by organ system with respiratory infections being the most common

During a total of 43 232 person-years follow-up, there were 1307 SI events (defined as hospital admission or death due to infection). The overall incidence rate (IR) of SI was 3.02 (95% CI 2.86–3.19) per 100 person years (Table 2). Respiratory infections, COVID-19 and sepsis/bacteraemia accounted for most of the SI events. The IR of SI events by organ class can be seen in Fig. 1. Of the total SI events, 41% were respiratory infections, followed by 15% COVID-19, 12% sepsis/bacteraemia, 10% genitourinary infections, 8% gastrointestinal infections, 7% skin infections, 4% for other infections, 1% bone infections, 0.23% cardiovascular infections, 0.15% for ear/nose and throat infections, while 0.08% were haematological, ophthalmology and nervous system–related infections.

**Table 2.** The risk of SI events in patients with RA in relation to initial treatment strategies and CS use (imputed analyses)

	Whole cohort	Any non- MTX strategy	MTX	No DMARD strategy compared with any non-MTX strategy	No steroid	Steroid
Number of patients	17 472	4540	**10 997**	**1935**	3721	**13 680**
Exposure (per 100 patient-years)	43 232	11 054	**27 500**	**4669**	9413	**33 689**
No. of SI events	1307	402	**725**	**180**	241	**1061**
IR of SI events (95% CI)	3.02 (2.86–3.19)	3.63 (3.29–4.00)	**2.63 (2.45–2.83)**	**3.85 (3.33–4.46)**	2.56 (2.25–2.90)	**3.14 (2.96–3.34)**
HR SI events, unadjusted (95% CI), *P*-value	N/A	Ref	**0.72 (0.63–0.82), *P* < 0.001**	**1.32 (1.18–1.57), *P* = 0.001**	Ref	**1.22 (1.07–1.38), *P* < 0.005**
HR SI events, age, gender adjusted (95% CI), *P*-value	N/A	Ref	**0.71 (0.63–0.80), *P* < 0.001**	**1.29 (1.00–1.52), *P* < 0.05**	Ref	**1.05 (0.93–1.19), *P* = 0.36**
HR SI events, fully adjusted^a^ (95% CI), *P*-value	N/A	Ref	**0.76 (0.67–0.86), *P* < 0.001**	**1.36 (1.15–1.59), *P* < 0.001**	Ref	**0.99 (0.87–1.12), *P* = 0.92**

The IR and HR of SI (admissions and mortality) in patients with early RA in relation to initial treatment strategies and CS use (imputed analyses). Any MTX strategy and no DMARD strategy were compared with any non-MTX strategy. Steroids compared with no steroids use. ^a^Fully adjusted are adjusted for age, gender, smoking status, social deprivation using IMD, comorbidity [diabetes mellitus, hypertension and lung disease, baseline disease severity (DAS28) and RF/CCP seropositivity]. HR: hazard ratio; IMD: Index of Multiple Deprivation; IR: incidence rate; SI: serious infection; DAS28: DAS based on 28 joint counts.

**Figure 2.**
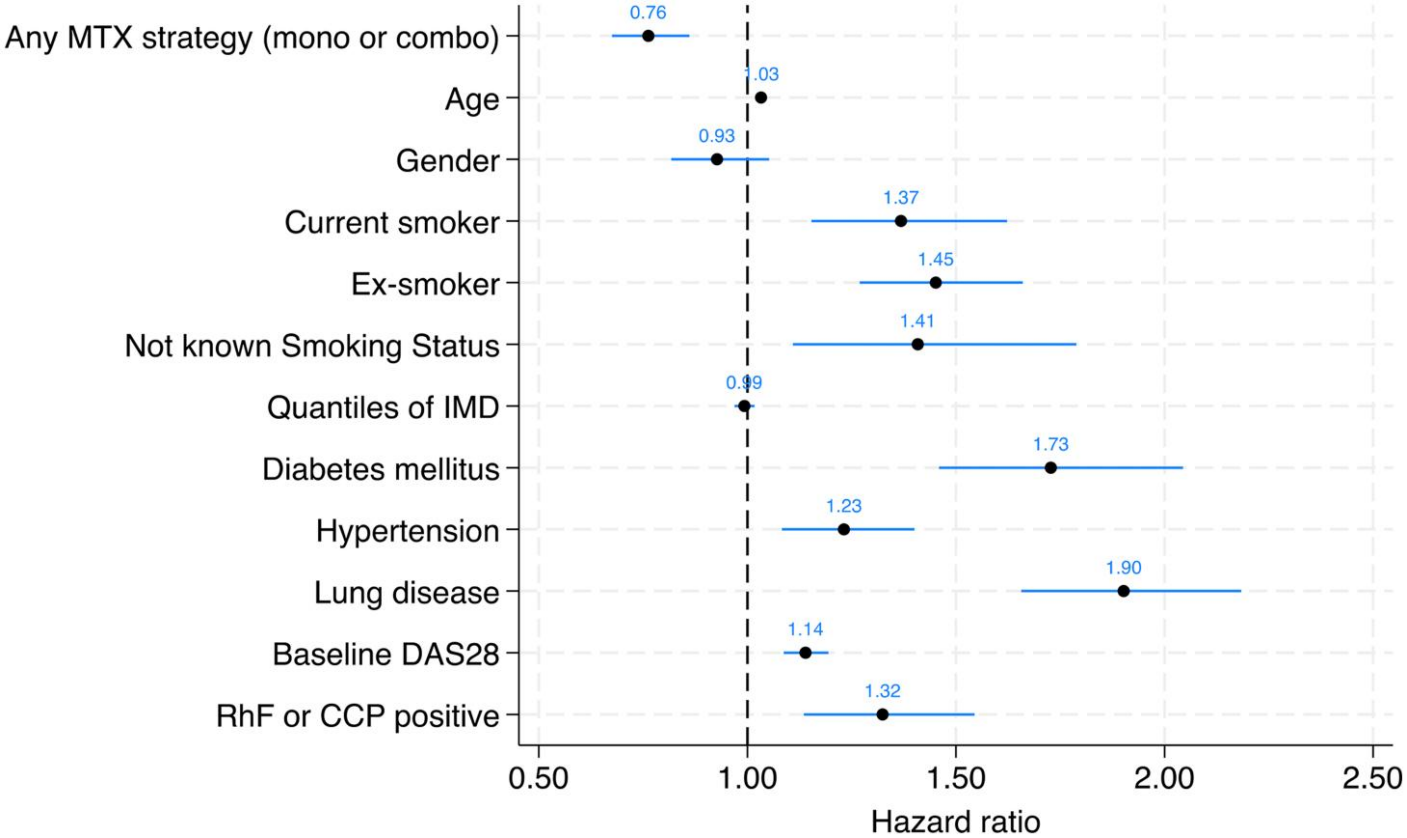
The hazards ratio of serious infections of methotrexate-based strategy in multivariable model

**Figure 3.**
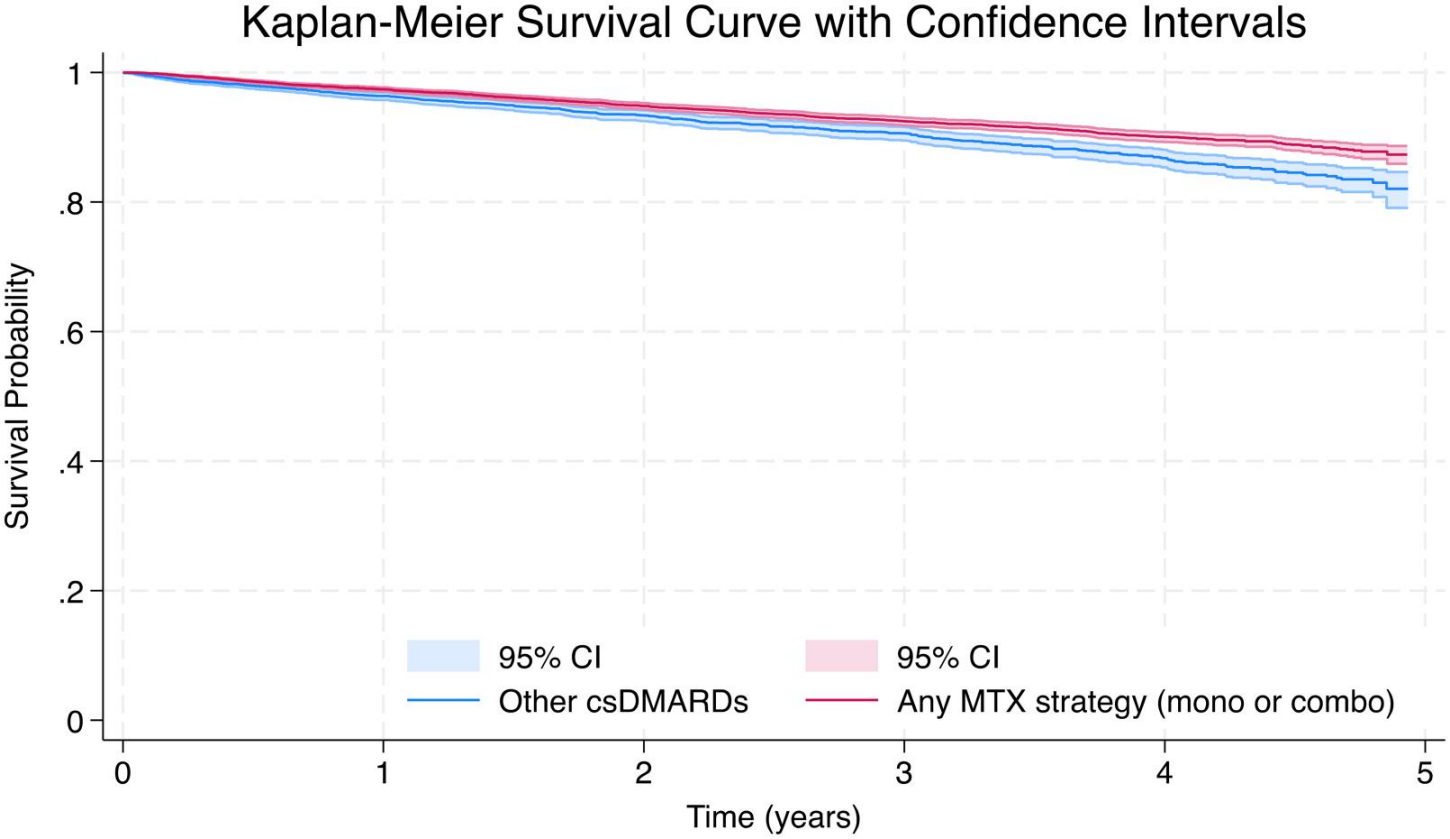
Five-year survival probability comparing methotrexate-based versus any other csDMARD strategies with confidence intervals

The IR and adjusted HR for SI related to csDMARD strategy can be seen in Table 2. The IR for SI was higher among those on non-MTX regimens (IR 3.63, 95% CI 3.29–4.0) compared with those on MTX (IR 2.63, 95% CI 2.45–2.83), corresponding to a reduced risk of SI in the MTX group compared with the non-MTX group [adjusted HR 0.76 (95% CI 0.67–0.86, *P* < 0.001)] (Fig. 2). Kaplan–Meier survival curve of csDMARDs SI can be seen in (Fig. 3). When we ran the sensitivity analysis examining the SI risk with csDMARDs monotherapy (Supplementary Table S4, available at *Rheumatology* online), MTX monotherapy remains the most commonly prescribed csDMARD and demonstrated the lowest incidence of SI events in this cohort. SSZ and LEF monotherapies showed higher infection rates. In Cox regression analyses, there was an excess risk of infection when compared with MTX monotherapy in the unadjusted model, although this did not persist after multivariate adjustment, suggesting potential confounding by indication. HCQ monotherapy showed low infection risk with no significant increase in hazards when compared with MTX monotherapy.

The IR and adjusted HR for SI according to CS use can be seen in Table 2. Patients who started CS had a numerically higher SI rate (IR 3.14, 95% CI 2.96–3.34) compared with those not on CS (IR 2.56, 95% CI 2.25–2.90); however, in adjusted models, CS use was not significantly associated with increased risk of SI (adjusted HR 0.99, 95% CI 0.87–1.12, *P* = 0.92) (Supplementary Fig. S2, available at *Rheumatology* online). Kaplan–Meier curve of CS SI are shown in (Supplementary Fig. S3, available at *Rheumatology* online).

Predictors of SI events in fully adjusted models across all treatment strategies can be seen in Table 3. In multivariable models, a number of variables were associated with an increased risk of SI: increasing age (adjusted HR 1.03 per 1 year increase, 95% CI 1.02–1.04), current smoking (adjusted HR 1.38, 95% CI 1.17–1.64) or past smoking (adjusted HR 1.41, 95% CI 1.24–1.59), and comorbidities [diabetes (adjusted HR 1.77, 95% CI 1.53–2.06), hypertension (adjusted HR 1.30, 95% CI 1.15–1.46), lung disease (adjusted HR 2.06, 95% CI 1.83–2.31)], higher baseline disease severity (adjusted HR 1.10 per 1 unit increased in DAS28, 95% CI 1.06–1.15) and seropositivity for RF (adjusted HR 1.57, 95% CI 1.06–1.44) predicted higher SI events. The association was not significant for CCP positive although the direction of effect was towards an increased risk (adjusted HR 1.14, 95% CI 0.93–1.38). This may relate to small number of patients being CCP positive (*n* = 1850; 10.6%). Asian ethnicity (compared with White ethnicity) was associated with few SI events (adjusted HR 0.60, 95% CI 0.44–0.82). Other ethnicities did not emerge as significant. Unadjusted and age/gender-adjusted predictor variable models are presented in Supplementary Table S5, available at *Rheumatology* online.

**Table 3.** Predictors of SI events in fully adjusted models (imputed analyses)

Predictor	HR	95% CI	*P*-value
Age	1.03	(1.02–1.04)	<0.001
Gender (female)	0.93	(0.83–1.05)	0.27
Ethnicity compared with White			
Black	0.99	(0.73–1.32)	0.95
Asian	0.60	(0.44–0.82)	0.001
Mixed	0.57	(0.21–1.53)	0.27
Other	0.46	(0.29–0.72)	0.001
More socially deprived (IMD quintile)	0.98	(0.94–1.03)	0.60
Smoking compared with never smoked			
Current smoker	1.38	(1.17–1.64)	<0.001
Ex-smoker	1.41	(1.24–1.59)	<0.001
Unknown smoking status	1.46	(1.16–1.85)	<0.005
Comorbidities			
Diabetes mellitus	1.77	(1.53–2.06)	<0.001
Hypertension	1.30	(1.15–1.46)	<0.001
Lung disease	2.06	(1.83–2.31)	<0.001
Disease characteristics			
Baseline DAS28	1.10	(1.06–1.15)	<0.001
RF positive	1.24	(1.06–1.44)	0.005
CCP positive	1.14	(0.93–1.38)	0.18
Double positive (RF and CCP)	1.57	(0.98–1.35)	0.07

Predictors of serious infections events (deaths and admissions) in fully adjusted models (imputed analyses) in patients with early RA. Presented model is adjusted for age, gender, smoking status, social deprivation using (IMD), comorbidity [diabetes mellitus, hypertension and lung disease, baseline disease severity (DAS28) and RF/CCP seropositivity]. SI: serious infection; DAS28: disease activity score at baseline based on 28 joint counts; HR: hazard ratio; IMD: Index of Multiple Deprivation.

### Infection-related mortality

Details on SI-related mortality are summarized in Supplementary Table S6, available at *Rheumatology* online. There were 311 SI-related mortality events over the study period. The overall IR of SI deaths was 0.69 (95% CI 0.61–0.77) per 100 person years.

To contextualize the data, SMR were calculated by comparing observed serious infections to expected infection-related mortality in the general population.

The overall SMR in our cohort was higher for RA patients compared with the general population (SMR 3.94, 95% CI 3.53–4.39). The increased mortality risk was more pronounced in males and younger patients. Details on the SMR by age and gender can be seen in Supplementary Fig. S4 and Table S7, available at *Rheumatology* online. A subanalysis was done on SMR excluding COVID-19 (SMR 7.19, 95% CI 6.41–8.04), with the highest SMR observed in the 50–64 years age group (Supplementary Fig. S5 and Table S8, available at *Rheumatology* online).

Analyses looking into treatment strategies and infection-related mortality revealed similar patterns to the SI analysis, with higher infection-related mortality in those on non-MTX-based regimens (IR 0.81, 95% CI 0.66–0.99) compared with those on MTX regimens (IR 0.57, 95% CI 0.49–0.66), though the adjusted HR was not statistically significant (adjusted HR 0.80, 95% CI 0.60–1.07). Similarly, patients starting CS had a higher mortality rate (IR 0.71, 95% CI 0.63–0.81) *vs* no CS (IR 0.59, 95% CI 0.46–0.77), but this was not significant in the adjusted model (adjusted HR 0.77, 95% CI 0.56–1.06) (presented in previously mentioned Supplementary Table S6, available at *Rheumatology* online).

Predictors of SI mortality are shown in Supplementary Table S9, available at *Rheumatology* online. Older age, smoking exposure, comorbidities, higher DAS28, and being RF and CCP positive were associated with higher SI mortality. Female gender was associated with fewer SI deaths compared with males. SI mortality did not emerge as significantly different between the different ethnicities. Unadjusted and age/gender adjusted models are also shown in (Supplementary Table S10, available at *Rheumatology* online).

Patients who did not receive a csDMARD at diagnosis had worse outcomes compared with who started on csDMARDs, in terms of both SI events (IR 3.85, 95% CI 3.33–4.46) and SI-related mortality (IR 1.10, 95% CI 0.84–1.44), though the latter was not significant (Table 2 and Supplementary Table S6, available at *Rheumatology* online).

## Discussion

This report quantifies the risk of SI in a large contemporary cohort in England and Wales of patients starting treatment for RA, offering insights into early treatment strategies using csDMARDs and CS. The overall IR of SI was 3.02 per 100 person years and SI-related mortality of 0.69 per 100 patient-years. Respiratory infections were the most common, consistent with previous reports on established RA [14, 15].

Although most guidelines advise limiting CS use in RA, their use is still common, with 30–60% of patients with RA remaining on long-term steroid therapy [16, 17]. In our analysis, unadjusted models showed an association between steroids and SI. It is likely that confounding factors, as increasing age, comorbidities and disease activity were driving this risk, as the association was no longer observed in adjusted models. Our previous network meta-analysis of randomized controlled trials (RCTs) in early RA did not identify a clear link between any specific initial treatment including steroids and an increased risk of infection, though the systematic review steroid data were mostly drawn from historic RCTs [18, 19].

Previous cohort studies in established RA suggest a dose and a duration-dependent risk of infections with steroids. Long-term use of high doses (>10 mg/day) has been associated with more than double the risk of SI [17, 20, 21]. A more recent cohort study using Swedish registry data on patients with long-term RA reported an increased incidence of SI with steroids with higher doses more than the lower doses and with more recent steroids exposure compared with past exposure [22]. A large systematic review on well-established RA recorded an increased risk of steroids infections in observational studies but this risk was not observed in RCTs, with reports on heterogeneity of exposures, outcome measures and confounder adjustments in the included observational studies [19].

Trials using low-dose CS suggested a modest risk of adverse events [23, 24], suggesting that the way CS are prescribed in early disease in England and Wales in our cohort (short courses of low doses with rapid tapering) may mitigate the infection risk. However, our result should be interpreted with several caveats, as assessment of steroids exposure was imperfect. The steroid data are captured at one time point, and we do not collect steroid dose in NEIAA. CS should still be used with caution especially with long-term use, and should not be considered risk-free.

MTX has historically been linked to interstitial lung disease [25], possibly explaining why patients with baseline lung disease were less likely to receive it (9% *vs* 17% for other csDMARDs). However, this association was recently challenged, with studies suggesting a reduced risk of RA-related lung disease with MTX [26, 27]. Studies examining infection risk with MTX are conflicting [17]—some report no increased risk, while others suggest a modest risk [28–30]. A large retrospective cohort in Canada included 27 710 patients with well-established RA with 162 710 patient-years of follow up, and reported a reduction in risk of mild infections in csDMARDs with no increased risk of serious infections (rate ratio 0.88, 95% CI 0.79–0.99) [31].

Similarly, a recently published RCT on cardiovascular inflammation reduction enrolled 9300 participants for secondary prevention of cardiovascular disease compared MTX *vs* placebo [32]. The trial showed no significant difference in SI rates (2.2 *vs* 2.5 per 100 person-years, *P* = 0.5) but indicated a slight increase in overall infection risk. Collectively, these findings suggest that MTX may slightly elevate the risk of non-serious infections while having minimal impact on severe infection risk. In our cohort, MTX was associated with a lower rate of serious infections compared with other DMARD strategies. While residual confounding may exist, our findings suggest that concerns about MTX increasing infection risk may be unfounded.

Patients not started on any DMARD had worse outcomes—untreated RA has been reported previously to contribute to poor health outcomes [33, 34]. The reasons why csDMARDs were not recorded in some patients within the first 3 months are not captured in NEIAA. This could reflect patient-level factors (such as comorbidities, frailty or patient choice) influencing the decision to start treatment or systemic issues (such as delays in scheduling education or treatment initiation) leading to delay in starting treatment. Further research is needed to clarify this relationship.

The risk of SI events was found to be increased with age, in patients who have/had a smoking exposure compared with to those who never smoked, and in those with comorbidities. The link between these variables were reported in other observational studies in individuals with established RA [35, 36]. We report that a one unit increase in baseline DAS28 score increases the risk of SI by 10%. These estimates are lower than those reported in well-established RA, with a DAS28 change of one unit during follow-up predicting a 27% increase in serious infection rates [37]. Seropositivity also predicted higher SI events in our cohort and this was also reported in the British Biologics Register [38]. Patients of Asian ethnicity were observed to have a lower rates of SI events compared with White patients. The reasons for this are unclear, possible explanations include differentials in healthcare-seeking behaviour, language barriers or access to services. Previous research has reported disparities in care access among ethnic minority groups [39, 40]. Further research is warranted to better understand this association.

The SMR for infection-related mortality including COVID-19 deaths was 3.94 (95% CI 3.53–4.39). These results are lower than the previous published papers on cause-specific SMRs, where they reported high SMRs for pneumonia only compared with the general population (SMR 5.2, 95% CI 2.3–10.3) [41, 42]. We included all ICD-10 codes for infections and sepsis in our study and it has been shown previously that sepsis mortality is high in patients with RA [43, 44].

A sensitivity analysis excluding COVID-19 deaths yielded a high SMR 7.19 (95% CI 6.41–8.04). This finding is consistent with our previous work on COVID-19 mortality risk in RA, which demonstrated that COVID-19 did not pose a substantially increased mortality risk in early RA compared with the general population [45]. The high SMR seen when excluding COVID-19 was consistent with an older study looking into septicaemia mortality risk in early RA [46]. Despite advancements in RA management, infection-related mortality persists [4, 46, 47].

The increased risk likely arises from a combination of factors, including immune dysregulation associated with RA, age-related comorbidities, increased exposure to infections, multidrug-resistant bacterial infections and treatment-related effects extending beyond the initial treatment strategy.

### Strengths and limitations

NEIAA represents the largest cohort of patients with early RA in England and Wales, providing a unique opportunity to evaluate the impact of initial treatment decisions on these contemporary patients. By linking NEIAA data to national hospital records, we ensured the robust identification of serious infection outcomes. We adopted a comprehensive approach to infection classification by including all relevant ICD-10 codes and stratifying serious infection risk by affected organ systems. Our analyses accounted for multiple confounder variables, thereby enhancing the validity of our findings.

Several limitations must be acknowledged. Treatment data on csDMARDs were only available at the time of diagnosis and up to 3 months. As a result, we were unable to account for patients who subsequently transitioned to biologic or targeted synthetic DMARDs or those who continued therapy. At the time of publication, UK guidelines require failure of two csDMARDs before biologics can be initiated. A recent analysis of the same cohort reported that 8.3% of patients were escalated to biologics within 12 months in NEIAA [48].

Steroids data was only available at baseline limiting assessment of long-term exposure. Unavailable data on treatment adherence, route of administration and steroid dosing further constrain interpretation.

Unmeasured confounders, as the type/severity of lung disease (e.g. asthma or chronic obstructive pulmonary disease) are not fully captured. The potential for ICD-10 coding misclassification could not be excluded, particularly in the case of COVID-19. Individuals with COVID-19 may have been assigned pneumonia-related ICD codes, and vice versa. Future research incorporating longitudinal treatment data and detailed patient-level validation of outcomes will be necessary to build on these findings and refine the understanding of infection risk in RA.

In summary, MTX-based regimens were associated with a reduced risk of serious infections compared with other csDMARDs strategies. Channelling bias due to residual confounding is likely part of this explanation, but our data still suggest that avoidance of MTX because of concerns surrounding serious infection risk are not strongly supported by evidence. The excess mortality from infections in patients with RA remains a significant concern, highlighting the ongoing need for infection prevention and management strategies in this population.

## Supplementary Material

keaf312_Supplementary_Data

## Data Availability

Data access requests can be made through the Healthcare Quality Improvement Partnership.

## References

[1] Cobb S , Anderson F, Bauer W. Length of life and cause of death in rheumatoid arthritis. New Engl J Med 1953;249:553–6. 10.1056/NEJM195310012491402 13087647

[2] Ozen G , Pedro S, England BR et al Risk of serious infection in patients with rheumatoid arthritis treated with biologic versus nonbiologic disease‐modifying antirheumatic drugs. ACR Open Rheumatology 2019;1:424–32. 31777822 10.1002/acr2.11064 PMC6858027

[3] Doran MF , Crowson CS, Pond GR et al Frequency of infection in patients with rheumatoid arthritis compared with controls: a population-based study. Arthritis Rheum 2002;46:2287–93. 10.1002/art.10524 12355475

[4] Franklin J , Lunt M, Bunn D et al Risk and predictors of infection leading to hospitalisation in a large primary-care-derived cohort of patients with inflammatory polyarthritis. Ann Rheum Dis 2007;66:308–12. 10.1136/ard.2006.057265 PMC1856002 16984941

[5] Burgers LE , Raza K, van der Helm-van Mil AH. Window of opportunity in rheumatoid arthritis—definitions and supporting evidence: from old to new perspectives. RMD Open 2019;5:e000870. 31168406 10.1136/rmdopen-2018-000870 PMC6525606

[6] Crossfield SSR , Buch MH, Baxter P et al Changes in the pharmacological management of rheumatoid arthritis over two decades. Rheumatology (Oxford) 2021;60:4141–51. 33404652 10.1093/rheumatology/keaa892 PMC8409998

[7] Ledingham J , Gullick N, Irving K et al; BSR and BHPR Standards, Guidelines and Audit Working Group. BSR and BHPR guideline for the prescription and monitoring of non-biologic disease-modifying anti-rheumatic drugs. Rheumatology 2017;56:865–8. 10.1093/rheumatology/kew479 28339817

[8] Fraenkel L , Bathon JM, England BR et al 2021 American College of Rheumatology guideline for the treatment of rheumatoid arthritis. Arthritis Care Res (Hoboken) 2021;73:924–39. 34101387 10.1002/acr.24596 PMC9273041

[9] Smolen JS , Landewé RBM, Bergstra SA et al EULAR recommendations for the management of rheumatoid arthritis with synthetic and biological disease-modifying antirheumatic drugs: 2022 update. Ann Rheum Dis 2023;82:3–18. 36357155 10.1136/ard-2022-223356

[10] Nice.org.uk. NICE Quality standard for rheumatoid arthritis in over 16s. Published 2020. https://www.nice.org.uk/guidance/qs33 (29 November 2022, date last accessed).

[11] rheumatology.org.uk. UK: British Society for Rheumatology/clinical annual report. [Updated 2022]. https://www.rheumatology.org.uk/Portals/0/Documents/Practice_Quality/Audit/NEIA/2022/NEIAA%20Fourth%20Annual%20Report_FINAL.pdf?ver=2022-10-13-110553-063 (29 November 2022, date last accessed).

[12] International Classification of Diseases 10th Revision. World Health Organization, 2019 https://icd.who.int/browse10/2019/en (15 September 2024, date last accessed).

[13] Office for National Statistics. The 21st century mortality files: deaths dataset. ONS. https://www.ons.gov.uk/peoplepopulationandcommunity/birthsdeathsandmarriages/deaths/datasets/the21stcenturymortalityfilesdeathsdataset/current (10 October 2024, date last accessed).

[14] Curtis JR , Patkar N, Xie A et al Risk of serious bacterial infections among rheumatoid arthritis patients exposed to tumor necrosis factor α antagonists. Arthritis Rheum 2007;56:1125–33. 17393394 10.1002/art.22504

[15] Rutherford AI , Subesinghe S, Hyrich KL et al Serious infection across biologic-treated patients with rheumatoid arthritis: results from the British Society for Rheumatology Biologics Register for Rheumatoid Arthritis. Ann Rheum Dis 2018;77:905–10. 29592917 10.1136/annrheumdis-2017-212825

[16] Haraoui B , Jovaisas A, Bensen WG et al Use of corticosteroids in patients with rheumatoid arthritis treated with infliximab: treatment implications based on a real-world Canadian population. RMD Open 2015;1:e000078. 10.1136/rmdopen-2015-000078 PMC4612699 26509071

[17] Riley TR , George MD. Risk for infections with glucocorticoids and DMARDs in patients with rheumatoid arthritis. RMD Open 2021;7:e001235. 10.1136/rmdopen-2020-001235 PMC7893655 33597206

[18] Adas MA , Allen VB, Yates M et al A systematic review and network meta-analysis of the safety of early interventional treatments in rheumatoid arthritis. Rheumatology 2021;60:4450–62. 34003970 10.1093/rheumatology/keab429 PMC8487311

[19] Dixon WG , Suissa S, Hudson M. The association between systemic glucocorticoid therapy and the risk of infection in patients with rheumatoid arthritis: systematic review and meta-analyses. Arthritis Res Ther 2011;13:R139. 10.1186/ar3453 PMC3239382 21884589

[20] Wilson JC , Sarsour K, Gale S et al Incidence and risk of glucocorticoid‐associated adverse effects in patients with rheumatoid arthritis. Arthritis Care Res 2019;71:498–511. 10.1002/acr.23611 29856128

[21] George MD Baker JF Winthrop K et al Risk for serious infection with low-dose glucocorticoids in patients with rheumatoid arthritis. Ann Intern Med 2020;173:870–8. 32956604 10.7326/M20-1594 PMC8073808

[22] Barbulescu A , Sjölander A, Delcoigne B et al Glucocorticoid exposure and the risk of serious infections in rheumatoid arthritis: a marginal structural model application. Rheumatology 2023;62:3391–9. 36821426 10.1093/rheumatology/kead083 PMC10547528

[23] Da Silva JA , Jacobs JW, Kirwan JR et al Safety of low dose glucocorticoid treatment in rheumatoid arthritis: published evidence and prospective trial data. Ann Rheum Dis 2006;65:285–93. 10.1136/ard.2005.038638 PMC1798053 16107513

[24] Boers M , Hartman L, Opris-Belinski D et al; GLORIA Trial Consortium. Low dose, add-on prednisolone in patients with rheumatoid arthritis aged 65+: the pragmatic randomised, double-blind placebo-controlled GLORIA trial. Ann Rheum Dis 2022;81:925–36. 35641125 10.1136/annrheumdis-2021-221957 PMC9209692

[25] Kremer JM , Alarcón GS, Weinblatt ME et al Clinical, laboratory, radiographic, and histopathologic features of methotrexate‐associated lung injury in patients with rheumatoid arthritis. A multicenter study with literature review. Arthritis Rheum 1997;40:1829–37. 10.1002/art.1780401016 9336418

[26] Dawson JK , Quah E, Earnshaw B et al Does methotrexate cause progressive fibrotic interstitial lung disease? A systematic review. Rheumatol Int 2021;41:1055–64. 33515067 10.1007/s00296-020-04773-4 PMC8079289

[27] Kim K , Woo A, Park Y et al Protective effect of methotrexate on lung function and mortality in rheumatoid arthritis-related interstitial lung disease: a retrospective cohort study. Ther Adv Respir Dis 2022;16:17534666221135314. 36346076 10.1177/17534666221135314 PMC9647291

[28] Lopez‐Olivo MA , Siddhanamatha HR, Shea B et al; Cochrane Musculoskeletal Group. Methotrexate for treating rheumatoid arthritis. Cochrane Database Syst Rev 2014;2014:CD000957. 10.1002/14651858.CD000957.pub2 PMC7047041 24916606

[29] Ibrahim A , Ahmed M, Conway R et al Risk of Infection with Methotrexate Therapy in Inflammatory Diseases: a Systematic Review and Meta-Analysis. Journal of Clinical Medicine 2018;8:15. 30583473 10.3390/jcm8010015 PMC6352130

[30] Solomon DH Glynn RJ Karlson EW et al Adverse effects of low-dose methotrexate. Ann Intern Med 2020;172:369–80. 32066146 10.7326/M19-3369 PMC7229518

[31] Lacaille D , Guh DP, Abrahamowicz M et al Use of nonbiologic disease-modifying antirheumatic drugs and risk of infection in patients with rheumatoid arthritis. Arthritis Rheum 2008;59:1074–81. 18668604 10.1002/art.23913

[32] Ridker PM , Everett BM, Pradhan A et al; CIRT Investigators. Low-dose methotrexate for the prevention of atherosclerotic events. New Engl J Med 2019;380:752–62. 10.1056/NEJMoa1809798 PMC6587584 30415610

[33] Nikiphorou E , Jacklin H, Bosworth A et al Disease impact of rheumatoid arthritis in patients not treated with advanced therapies; survey findings from the National Rheumatoid Arthritis Society. Rheumatol Adv Pract 2021;5:rkaa080. 10.1093/rap/rkaa080 PMC8314206 34322656

[34] Scott DL , Wolfe F, Huizinga TWJ. Rheumatoid arthritis. Lancet 2010;376:1094–108. 20870100 10.1016/S0140-6736(10)60826-4

[35] Mehta B , Pedro S, Ozen G et al Serious infection risk in rheumatoid arthritis compared with non-inflammatory rheumatic and musculoskeletal diseases: a US national cohort study. RMD Open 2019;5:e000935. 31245055 10.1136/rmdopen-2019-000935 PMC6560658

[36] Jani M , Barton A, Hyrich K. Prediction of infection risk in rheumatoid arthritis patients treated with biologics: are we any closer to risk stratification? Curr Opin Rheumatol 2019;31:285–92. 10.1097/BOR.0000000000000598 PMC6443047 30789850

[37] Emery P , Gallo G, Boyd H et al Association between disease activity and risk of serious infections in subjects with rheumatoid arthritis treated with etanercept or disease-modifying anti-rheumatic drugs. Clin Exp Rheumatol 2014;32:653–60. 25190189

[38] Subesinghe S , Rutherford AI, Byng-Maddick R et al Recurrent serious infections in patients with rheumatoid arthritis—results from the British Society for Rheumatology Biologics Register. Rheumatology 2018;57:651–5. 29340619 10.1093/rheumatology/kex469

[39] Ajayi Sotubo O. A perspective on health inequalities in BAME communities and how to improve access to primary care. Future Healthc J 2021;8:36–9. 33791458 10.7861/fhj.2020-0217 PMC8004339

[40] Robinson A , Elarbi M, Todd A et al A qualitative exploration of the barriers and facilitators affecting ethnic minority patient groups when accessing medicine review services: perspectives of healthcare professionals. Health Expect 2022;25:628–38. 10.1111/hex.13410 PMC8957739 34951087

[41] Barrett O , Abramovich E, Dreiher J et al Short-and long-term mortality due to sepsis in patients with rheumatoid arthritis. Rheumatol Int 2017;37:1021–6. 28286904 10.1007/s00296-017-3694-5

[42] Black RJ , Lester S, Tieu J et al Mortality estimates and excess mortality in rheumatoid arthritis. Rheumatology 2023;62:3576–83. 10.1093/rheumatology/kead106 PMC10629787 36919770

[43] Curtis JR , Xie F, Chen L et al The comparative risk of serious infections among rheumatoid arthritis patients starting or switching biological agents. Ann Rheum Dis 2011;70:1401–6. 21586439 10.1136/ard.2010.146365 PMC3128235

[44] Krasselt M , Baerwald C, Petros S et al Mortality of sepsis in patients with rheumatoid arthritis: a single-center retrospective analysis and comparison with a control group. J Intensive Care Med 2021;36:766–74. 32249644 10.1177/0885066620917588 PMC8165740

[45] Adas MA , Russell MD, Cook E et al COVID-19 admissions and mortality in patients with early inflammatory arthritis: results from a UK national cohort. Rheumatology (Oxford) 2023;62:2979–88. 36645234 10.1093/rheumatology/kead018 PMC10473194

[46] Young A , Koduri G, Batley M et al; Early Rheumatoid Arthritis Study (ERAS) Group. Mortality in rheumatoid arthritis. Increased in the early course of disease, in ischaemic heart disease and in pulmonary fibrosis. Rheumatology 2007;46:350–7. 16908509 10.1093/rheumatology/kel253

[47] Housden MM , Bell G, Heycock CR et al How to reduce morbidity and mortality from chest infections in rheumatoid arthritis. Clin Med (Lond) 2010;10:326–9. 20849003 10.7861/clinmedicine.10-4-326 PMC4952158

[48] Russell MD , Yang Z, Dooley N et al Temporal and regional variation in the use of biologic and targeted synthetic DMARDs for rheumatoid arthritis: a nationwide cohort study. Rheumatology 2025;64:2432–41. 10.1093/rheumatology/keae607 PMC12048046 39485485

