# Supplementary material for: Risk of infection in patients with early inflammatory arthritis: results from a large UK prospective observational cohort study
Source: Rheumatology (Oxford). 2025 Jun 5;64(11):5647–55. doi: 10.1093/rheumatology/keaf312 (PMC12596073; doi:10.1093/rheumatology/keaf312)
Supplement: keaf312_Supplementary_Data [file keaf312_supplementary_data.docx]

# **Appendix**

**Tables:**

[Supplementary Table S1: ICD10 codes for infections 2](#_Toc199769268)

[Supplementary Table S2: Baseline characteristics by adjuvant steroid therapy 5](#_Toc199769269)

[Supplementary Table S3: missing data and imputation details 6](#_Toc199769270)

[Supplementary Table S4: The risk of serious infections events in patients with RA in relation to csDMARDs monotherapy (imputed analyses) 7](#_Toc199769271)

[Supplementary Table S5: Unadjusted and age/gender adjusted models for SI events (main outcome) predictor model, (imputed analyses) 10](#_Toc199769272)

[Supplementary Table S6: SI mortality (secondary outcome) in relation to treatment strategies and corticosteroids use (imputed analyses) 11](#_Toc199769273)

[Supplementary Table S7: SMR of serious infections related mortality (Including COVID -19) 12](#_Toc199769274)

[Supplementary Table S8: SMR of serious infections related mortality (Excluding COVID -19) 13](#_Toc199769275)

[Supplementary Table S9: Predictors of SI related deaths (secondary outcome) in fully adjusted models (imputed analyses) 14](#_Toc199769276)

[Supplementary Table S10: Predictors of SI related deaths (secondary outcome) in unadjusted and age/gender adjusted models (imputed analyses) 15](#_Toc199769277)

**Figures:**

[Supplementary Figure S1: Cohort diagram chart 4](#_Toc199769281)

[Supplementary Figure S2: Hazards ratios (95%CI) of serious infections events in according to corticosteroids with estimates for covariates 8](#_Toc199769282)

[Supplementary Figure S3: Kaplan- Meier survival curve of SI events according to corticosteroids use 9](#_Toc199769283)

[Supplementary Figure S4: SMR of serious infections related mortality (Including COVID -19) 12](#_Toc199769284)

[Supplementary Figure S5: SMR of serious infections related mortality (Excluding COVID -19) 13](#_Toc199769285)

**Supplementary Table S1: ICD10 codes for infections**

| **Infection site** | **ICD-10 (2019 version)** |
| --- | --- |
| Nervous system | G000, G001, G002, G003, G008, G009, G01, G020, G021, G042, G050, G051, G052, G060, G061, G062, G07, G08, G630, A170, A171, A171, A178, A179, A800, A801, A802, A803, A804, A805, A809, A810, A811, A812, A818, A819, A820, A821, A822, A823, A824, A825, A829, A830, A831, A832, A833, A834, A835, A836, A837, A838, A839, A840, A841, A848, A849, A850, A851, A852, A858, A859, A860, A868, A869, A870, A871, A872, A878, A879, A880, A888, A89 |
| Endocrine | E033, E060, E321, E350, E351, A187 |
| Blood, blood forming organs and lymph nodes | D733, D762, A182 |
| Eyes | H000, H030, H031, H061, H130, H131, H190, H191, H192, H220, H230, H428, H440, H441, H451, H480, H481, H580, H588, A185 |
| Ear, Nose and throat (ENT) | H600, H601, H602, H603, H620, H621, H622, H623, H650, H652, H653, H660, H661, H662, H663, H664, H670, H671, H700, H701, H730, H731, H750, H940, A186 |
| Cardiovascular system | I301, I320, I321, I330, I410, I411, I412, I430, I520, I521, I681, I790, I791, I980, I981 |
| Respiratory system | J00, J010, J011, J012, J013, J014, J018, J020, J028, J029, J030, J038, J039, J040, J041, J042, J050, J051, J060, J068, J069, J09, J100, J101, J108, J110, J111, J118, J120, J121, J122, J123, J128, J129, J13, J14, J150, J151, J152, J153, J154, J155, J156, J157, J158, J159, J160, J168, J170, J171, J172, J173, J178, J180, J181, J182, J188, J189, J200, J201, J202, J203, J204, J205, J206, J207, J208, J209, J210, J211, J218, J219, J220, J311, J320, J321, J322, J323, J324, J328, J329, J340, J360, J370, J371, J390, J391, J393, J399, J400, J410, J411, J418, J650, J850, J851, J852, J853, J860, J869, J987, J998, A150, A151, A153, A154, A155, A156, A157, A158, A159, A16, A160, A161,A162, A163, A164, A165, A167, A168, A169, A188, A19, A190, A191, A192, A198, A199, B59, B441,B440,B449,B59 |
| Digestive system | K046, K047, K050, K051, K052, K113, K122, K200, K230, K231, K570, K572, K574, K578, K610, K611, K612, K613, K614, K630, K650, K670, K671, K672, K673, K678, K750, K770, K750, K810, K871, K930, K931, A00, A000, A001, A009, A01, A010, A011, A012, A013, A014, A02, A020, A021, A022, A028, A029, A03, A030, A031, A032, A033, A038, A039, A04, A040, A041, A042, A043, A044, A045, A046, A047, A048, A049, A05, A050, A051, A052, A053, A054, A058, A059, A06, A060, A061, A062, A063, A064, A065, A066, A067, A068, A069, A07, A070, A071, A072, A073, A07.8, A07.9, A08, A08.0, A08.1, A08.2, A08.3, A08.4, A08.5, A09, A09, A090,A099, A183, B150, B159, B160, B161, B162, B169, B170, B17, B1710, B1711, B172, B178, B179, B18, B180, B181, B182, B188, B189, B19,B190, B199 |
| Skin and subcutaneous tissue | L00, L010, L011, L020, L021, L022, L023, L024, L028, L029, L030, L031, L032, L038, L039, L040, L041, L042, L043, L048, L049, L050, L080, L081, L088, L089, L998, A184 |
| Bone and joints | M000, M001, M002, M008, M009, M010, M011, M013, M014, M015, M016, M018, M023, M030, M031, M032, M036, M600, M630, M631, M632, M650, M651, M680, M710, M711, M728, M730, M731, M860, M861, M862, M863, M864, M865, M866, M868, M869, M900, M901, M902, A180 |
| Genitourinary system | N080, N10, N11, N136, N151, N159, N160, N220, N290, N291, N308, N330, N338, N340, N351, N370, N390, N412, N431, N450, N481, N482, N499, N510, N511, N512, N518, N61, N700, N701, N709, N730, N731, N732, N734, N735, N738, N739, N740, N741, N743, N744, N751, N764, N770, N771, A181 |
| Reproductive, pregnancy, delivery and puerperium | O080, O230, O231, O232, O233, O234, O235, O239, O264, O411, O850, O860, O861, O862, O863, O868, O910, O911, O912, O980, O981, O982, O983, O984, O985, O986, O987, O988, O989 |
| Septicaemia | A327, A400, A401, A402, A403, A408, A409, A410, A411, A412, A413, A414, A415, A418, A419, A420, A421, A422, A427, A428, A429, A430, A431, A438, A439, A440, A441, A448, A449, A480, A481, A482, A483, A484, A488, A490, A491, A492, A493, A498, A499, A391, A392, A393, A394, A395, A398, A399 |
| Other infections | A200, A201, A202, A203, A207, A208, A209, A210, A211, A212, A213, A217, A218, A219, A220, A221, A222, A227, A228, A229, A230, A231, A232, A233, A238, A239, A240, A241, A242, A243, A244, A245, A248, A249, A250, A251, A252, A253, A254, A255, A259, A260, A267, A268, A269, A270, A278, A279, A280, A281, A282, A288, A289, A300, A301, A302, A303, A304, A310, A311, A312, A318, A319, A320, A321, A322, A327, A328, A329, A330, A331, A332, A338, A339, A340, A341, A342, A343, A344, A345, A348, A349, A350, A351, A352, A358, A359, A360, A361, A362, A363, A368, A369, A370, A371, A378, A379, A380, A388, A389, A390, A391, A392, A398, A399, A400, A401, A402, A403, A408, A409, A410, A411, A412, A413, A414, A415, A418, A419, A420, A421, A422, A423, A427, A428, A429, A430, A431, A432, A438, A439, A440, A441, A448, A449, A460, A467, A468, A469, A480, A481, A482, A483, A484, A488, A489, A490, A491, A492, A498, A499, A500, A501, A502, A503, A504, A505, A506, A507, A509, A510, A511, A512, A513, A514, A515, A519, A520, A521, A522, A523, A527, A528, A529, A530, A531, A538, A539, A540, A541, A542, A543, A544, A545, A546, A548, A549, A550, A551, A552, A553, A554, A559, A560, A561, A562, A563, A568, A569, A570, A571, A578, A579, A580, A588, A589, A590, A591, A592, A593, A594, A598, A599, A600, A601, A602, A603, A608, A609, A630, A638, A639, A640, A641, A642, A648, A649, A650, A651, A652, A659, A660, A661, A662, A663, A668, A669, A670, A671, A672, A673, A679, A680, A688, A689, A690, A691, A698, A699, A700, A701, A702, A708, A709, A710, A711, A712, A718, A719, A740, A748, A749, A750, A751, A752, A753, A759, A770, A771, A772, A773, A774, A775, A778, A779, A780, A788, A789, A790, A791, A798, A799,  A920, A921, A922, A923, A924, A928, A929, A930, A931, A932, A933, A934, A935, A936, A937, A938, A939, A940, A941, A942, A948, A949, A950, A951, A952, A958, A959, A960, A961, A968, A969, A970, A971, A978, A979, A980, A981, A982, A983, A984, A985, A988, A989, A99, B000, B001, B002, B003, B004, B005, B007, B008, B009, B010, B011, B012, B018, B019, B020, B021, B022, B023, B027, B028, B029, B030, B04, B05, B050, B051, B052, B053, B054, B058, B059, B60, B060, B068, B069, B07, B070, B080, B081, B082, B083, B084, B085, B088, B09  B20, B200, B201, B202, B203, B204, B205, B206, B207, B208, B209, B21, B210, B211,B212, B213, B217,B218, B219, B22, B220, B221, B222, B227, B23, B230, B231, B232, B238, B24, B25, B250, B251, B252, B258, B259, B26, B260, B261, B262, B263, B268, B269, B27, B270, B271, B278, B279, B30, B300, B301, B302, B303, B308 B309, B33, B330, B331, B332, B333, B334, B338, B34, B340, B341,B342, B343, B344, B348, B349, B35, B350, B351, B352, B353,B354, B355, B356, B358, B359, B36, B360, B361, B362, B363, B368, B369, B370, B371, B372, B373, B374, B375, B376, B377, B378, B379, B380, B381, B382, B383, B384, B387, B388, B389, B390, B391, B392, B393, B394, B395, B399, B400, B401, B402, B403, B407, B408, B409, B410, B417 -B419, B420, B421, B427, B428, B429, B430, B431, B432, B438, B439, B451, B452, B453, B457, B458, B459, B461, B462, B463, B464, B465, B468, B469, B470, B471, B479, B480, B481, B482, B483, B484, B485, B487, B488, B49, B500, B508, B509, B510, B518, B519, B520, B528, B529, B530, B531, B538, B54, B550, B551, B552, B559, B560, B561, B569, B570, B571, B572, B573, B573, B575, B580, B581, B582, B583, B588, B589, B600, B601, B602, B608, B64, B650, B651, B652, B653, B658, B659, B660, B661, B662, B663, B664, B665, B668, B669, B670, B671, B672, B673, B674, B675, B676, B677, B678, B679, B680, B681, B689, B690, B691, B698, B699, B700, B701, B710, B711, B718, B719, B72, B73, B740, B741, B742, B743, B744, B748, B749, B75, B760, B761, B768, B769, B770, B778, B779, B780, B781, B787, B789, B79, B80, B810, B811, B812, B813, B814, B818, B820, B829, B830, B831, B832, B833, B834, B838, B839, B850, B851, B852, B853, B854, B86, B870, B871, B872, B873, B874, B878, B879, B880, B881, B882, B883, B888, B889, B89, B900, B901, B902, B908, B909, B91, B92, B940, B941, B942, B948, B949, B950, B951, B952, B953, B954, B955, B956, B957, B958, B960, B961, B962, B963, B964, B965, B966, B967, B968, B970, B971, B972, B973, B974, B975, B976, B2977, B978, B980, B981, B99 |

International disease classification (ICD10) codes for every infection were extracted using the world health organisation (WHO) ICD classification using the 2019 online version <https://icd.who.int/browse10/2019/en#/P35.0>

**Supplementary Figure S1: Cohort diagram chart**

NEIAA patients from (May 2018- April 2023) with suspected inflammatory arthritis

n= 67,720

Patients with confirmed inflammatory arthritis

n= 25,308

Patients with full treatment data by three months

n= 17,472

Patients with confirmed rheumatoid arthritis diagnosis

n= 17,803

Linked to NHS Digital

NEIAA, National Early Inflammatory Arthritis Audit; NHS, National Health Services.

**Supplementary Table S2: Baseline characteristics by adjuvant steroid therapy**

| **Variables** | **Total with steroids data** | **Started adjuvant steroids** | **Not started adjuvant steroids** |
| --- | --- | --- | --- |
|  | **N= 17,401** | **N=13,680** | **N=3,721** |
| **Age: mean (SD)** | 59 (15) | 60 (15) | 56 (15) |
| **Age band: n (%)** |  |  |  |
| 16- | 323 (1.9%) | 225 (1.6%) | 98 (2.6%) |
| 25- | 1,831 (10.5%) | 1,337 (9.8%) | 494 (13.3%) |
| 40- | 8,133 (46.7%) | 6,240 (45.6%) | 1,893 (50.9%) |
| 65- | 4,035 (23.2%) | 3,261 (23.8%) | 774 (20.8%) |
| 75- | 3,079 (17.7%) | 2,617 (19.1%) | 462 (12.4%) |
| **Gender: n (%)** |  |  |  |
| Male | 6,372 (36.6%) | 5,124 (37.5%) | 1,248 (33.5%) |
| Female | 11,029 (63.4%) | 8,556 (62.5%) | 2,473 (66.5%) |
| **Smoking status: n (%)** |  |  |  |
| Current smoker | 3,320 (19.1%) | 2,611 (19.1%) | 709 (19.1%) |
| Ex-smoker | 5,031 (28.9%) | 4,025 (29.4%) | 1,006 (27.0%) |
| Never smoked | 7,449 (42.8%) | 5,840 (42.7%) | 1,609 (43.2%) |
| Not known | 1,601 (9.2%) | 1,204 (8.8%) | 397 (10.7%) |
| **Social deprivation (IMD quantiles): n (%)** |  |  |  |
| 1 (most deprived) | 2,878 (17.4%) | 2,293 (17.7%) | 585 (16.5%) |
| 2 | 3,420 (20.7%) | 2,610 (20.1%) | 810 (22.9%) |
| 3 | 3,476 (21.1%) | 2,699 (20.8%) | 777 (21.9%) |
| 4 | 3,244 (19.7%) | 2,602 (20.1%) | 642 (18.1%) |
| 5 (least deprived) | 3,480 (21.1%) | 2,750 (21.2%) | 730 (20.6%) |
| **Ethnicity: n (%)** |  |  |  |
| White | 14,760 (84.8%) | 11,784 (86.1%) | 2,976 (80.0%) |
| Black | 462 (2.7%) | 342 (2.5%) | 120 (3.2%) |
| Asian | 1,368 (7.9%) | 1,015 (7.4%) | 353 (9.5%) |
| Mixed | 85 (0.5%) | 60 (0.4%) | 25 (0.7%) |
| Other | 550 (3.2%) | 380 (2.8%) | 170 (4.6%) |
| Not known | 176 (1.0%) | 99 (0.7%) | 77 (2.1%) |
| **Comorbidities: n (%)** |  |  |  |
| Diabetes mellitus | 1,658 (10%) | 1,289 (9%) | 369 (10%) |
|  |  |  |  |
| Hypertension | 3,661 (21%) | 2,970 (22%) | 691 (19%) |
|  |  |  |  |
| Lung disease | 1,981 (11%) | 1,613 (12%) | 368 (10%) |
|  |  |  |  |
| **Serostatus: n (%)** |  |  |  |
| Seronegative | 5,353 (30.8%) | 4,309 (31.5%) | 1,044 (28.1%) |
| RhF positive | 2,670 (15.3%) | 2,111 (15.4%) | 559 (15.0%) |
| CCP positive | 1,840 (10.6%) | 1,373 (10.0%) | 467 (12.6%) |
| Double positive (RhF and CCP) | 7,538 (43.3%) | 5,887 (43.0%) | 1,651 (44.4%) |
|  |  |  |  |
| **Duration Symptoms: n (%)** |  |  |  |
| <1 month | 1,378 (8.0%) | 1,145 (8.4%) | 233 (6.3%) |
| 1-3 months | 5,886 (34.0%) | 4,803 (35.3%) | 1,083 (29.5%) |
| 3-6 months | 4,156 (24.0%) | 3,318 (24.4%) | 838 (22.8%) |
| 6-12 months | 3,204 (18.5%) | 2,454 (18.0%) | 750 (20.4%) |
| > 12 months | 2,666 (15.3%) | 1,894 (13.8%) | 772 (20.7%) |
|  |  |  |  |
| **Baseline DAS28: mean (SD)** | 4.9 (1.4) | 5.1 (1.4) | 4.1 (1.4) |
| **ESR mm/hr: median (IQR)** | 27.0 (12.0,45.0) | 29.0 (13.5,47.0) | 20.0 (8.0,37.0) |
| **CRP mg/L: median (IQR)** | 11.0 (4.0,30.0) | 13.0 (5.0,33.0) | 7.0 (3.0,18.0) |

Total cohort is 17,472, missing data for adjuvant steroids n=71

CCP, cyclic citrullinated peptide; CRP, C-reactive protein; DAS28, disease activity score based on 28 joint counts; ESR, erythrocyte sedimentation rate; IMD, index of multiple deprivation; RhF, rheumatoid factor.

**Supplementary Table S3: missing data and imputation details**

| **Variable** | **Number of missing data**  **n (%)** |
| --- | --- |
| **Age** | 0 (0%) |
| **Gender** | 0 (0%) |
| **Smoking** | 0 (0%) |
| **Ethnicity** | 0 (0%) |
| **IMD** | 903 (5.1%) |
| **Diabetes** | 10 (0.05%) |
| **Hypertension** | 10 (0.05%) |
| **Lung disease** | 10 (0.05%) |
| **RF or CCP Seropositivity** | 829 (4.74%) |
| **Baseline DAS28** | 1,013 (5.79%) |
| **Adjuvant steroids** | 71(0.40%) |

Total number of included patients n=17,472 with full csDMARDs data. Imputation model included comorbidities, social deprivation (IMD), baselineDAS28, and seropositivity were imputed, multiple imputation model using chained equations (20 imputed datasets) was used for hazards ratio analyses.

All missing data were imputed regardless of the reason/s they were missing. The following baseline variables with complete data where data were utilised for the imputation: age; gender; smoking status, start (time of diagnosis or May 2018) and failure time (time of first infection or death).

The study outcome was steroids risk and missingness in steroids data was not imputed.

CCP, cyclic citrullinated peptide; DAS28, disease activity score based on 28 joint counts; IMD, index of multiple deprivation; RhF, rheumatoid factor.

**Supplementary Table S4**: **The risk of serious infections events in patients with RA in relation to csDMARDs monotherapy (imputed analyses)**

|  | **Whole cohort** | **MTX monotherapy** | **HCQ monotherapy** | **SSZ monotherapy** | **Other csDMARD monotherapy including leflunomide ^$^** |
| --- | --- | --- | --- | --- | --- |
| **Number of patients** | 17,472 | 8,846 | 2,649 | 1,536 | 72 |
| **Exposure (per 100 patient years)** | 43,232 | 21,471 | 6,576 | 3,659 | 0.192 |
| **No. of SI events** | 1307 | 594 | 197 | 153 | 11 |
| **IR of SI events, (95% CI)** | 3.02 (2.86-3.19) | 2.76 (2.55-2.99) | 2.99 (2.60-3.44) | 4.18 (3.56-4.56) | 5.73 (3.17-10.35) |
| **HR SI events unadjusted**  **(95% CI), p value** | N/A | Ref | 0.99 (0.84-1.16), p=0.92 | 1.43 (1.22-1.68), p<0.001 | 1.92 (1.00-3.70), p=0.04 |
| **HR SI events Age, gender adjusted (95% CI), p value** | N/A | Ref | 1.08 (0.93-1.26), p=0.28 | 1.29 (1.10-1.51), p=001 | 1.69 (0.89-3.19), p=0.10 |
| **HR SI events *fully adjusted (95% CI), p value** | N/A | Ref | 1.09 (0.93-1.27), p=0.27 | 1.15 (0.97-1.36), p=0.10 | 1.37 (0.80-2.35), p=0.24 |

csDMARDs monotherapies SI risk (admission and related mortality) is presented in this table, **all combination therapies n= 2,434 are not presented above as it is difficult to interpret SI risk with overlapping DMARDs. No DMARD n= 1,935 strategy SI risk and hazards are presented in the main table.** Hazards ratios in this table are compared to methotrexate monotherapy.

^$^ Details on the other csDMARD category are not captured in NEIAA.

*Fully adjusted are adjusted for age, gender, smoking status, social deprivation using (IMD), comorbidity (DM, HTN and lung disease, baseline disease severity (DAS28) and RhF/CCP seropositivity.

**Supplementary Figure S2: Hazards ratios (95%CI) of serious infections events in according to corticosteroids with estimates for covariates**

Hazards ratios and (95%CI) according to corticosteroid use in fully adjusted model, estimating the risk of serious infection event (admission and mortality) in patients with early RA. Age (by year), IMD (by quantile), Baseline DAS28 (by each unit increase).

CCP, cyclic citrullinated peptide; DAS28, disease activity score at baseline based on 28 joint counts; IMD, index of multiple deprivation; RhF, rheumatoid factor.

**Supplementary Figure S3: Kaplan- Meier survival curve of SI events according to corticosteroids use**

This is a Kaplan-Meier Survival Curve in patients with early RA showing the survival probability from serious infections events, over a five-year period according to corticosteroids use at baseline. Both groups show a gradual decline in survival probability over time, with overlapping confidence intervals suggesting similar survival trends between the groups during the 5-year follow-up period.

RA, rheumatoid arthritis.

**Supplementary Table S5: Unadjusted and age/gender adjusted models for SI events (main outcome) predictor model, (imputed analyses)**

| **Predictor** | **Hazards ratio (HR)** | **95% CI** | **P value** | **Hazards ratio (HR)** | **95% CI** | **P value** |
| --- | --- | --- | --- | --- | --- | --- |
| **Un-adjusted model** | | | | **Age-Gender adjusted models** | | |
| **Age** | 1.04 | (1.03-1.04) | <0.001 | 1.04 | (1.03-1.04) | <0.001 |
| **Gender (female)** | 0.73 | (0.65-0.82) | <0.001 | 0.84 | (0.75-0.94) | <0.05 |
| **Ethnicity compared to White:** |  |  |  |  |  |  |
| Black | 0.99 | (0.73-1.32) | 0.95 | 1.36 | (0.99-1.87) | 0.05 |
| Asian | 0.60 | (0.44-0.82) | <0.005 | 0.93 | (0.68-1.26) | 0.64 |
| Mixed | 0.57 | (0.21-1.53) | 0.27 | 0.87 | (0.34-2.24) | 0.78 |
| Other | 0.46 | (0.29-0.72) | <0.05 | 0.58 | (0.37-0.92) | 0.02 |
| **More socially deprived (IMD quantile)** | 0.98 | (0.94-1.02) | 0.43 | 1.01 | (0.97-1.06) | 0.38 |
| **Smoking compared to never smoked:** |  |  |  |  |  |  |
| Current Smoker | 1.35 | (1.15-1.59) | <0.001 | 1.59 | (1.36-1.88) | <0.001 |
| Ex-Smoker | 1.80 | (1.60-2.03) | <0.001 | 1.56 | (1.39-1.76) | <0.001 |
| Not Known Smoking status | 1.56 | (1.24-1.97) | <0.001 | 1.42 | (1.13-1.79) | <0.005 |
| **Comorbidities:** |  |  |  |  |  |  |
| Diabetes mellitus | 2.27 | (1.95-2.63) | <0.001 | 1.80 | (1.54-2.09) | <0.001 |
| Hypertension | 1.93 | (1.72-2.16) | <0.001 | 1.29 | (1.15-1.45) | <0.001 |
| Lung disease | 2.79 | (2.47-3.15) | <0.001 | 2.25 | (2.00-2.53) | <0.001 |
| **Disease characteristics:** |  |  |  |  |  |  |
| Baseline DAS28 | 1.17 | (1.12-1.22) | <0.001 | 1.11 | (1.07-1.16) | <0.001 |
| RhF positive | 1.16 | (1.00-1.35) | 0.04 | 1.32 | (1.13-1.53) | <0.001 |
| CCP positive | 0.93 | (0.77-1.13) | 0.50 | 1.15 | (0.95-1.40) | 0.13 |
| Double positive (RhF and CCP) | 1.00 | (0.86-1.16) | 0.93 | 1.27 | (1.08-1.50) | <0.005 |

Predictors of serious infections events (deaths and admissions) in unadjusted and age/gender adjusted models (imputed analyses) in patients with early RA.

Presented model is adjusted for age, gender, smoking status, social deprivation using (IMD), comorbidity (DM, HTN and lung disease, baseline disease severity (DAS28) and RhF/CCP seropositivity.

CCP, cyclic citrullinated peptide; DAS28, disease activity score at baseline based on 28 joint counts; HR: hazards ratio; IMD, index of multiple deprivation; RhF, rheumatoid factor.

**Supplementary Table S6: SI mortality (secondary outcome) in relation to treatment strategies and corticosteroids use (imputed analyses)**

|  | **Whole cohort** | **Any non- Methotrexate strategy** | **Any Methotrexate strategy** | **No DMARD**  **Compared to any non-methotrexate strategy** | **No Steroid** | **Steroid** |
| --- | --- | --- | --- | --- | --- | --- |
| **Number of patients** | 17,472 | 4,540 | **10,997** | **1,935** | 3,721 | **13,680** |
| **Exposure (per 100 patient years)** | 44,910 | 11,544 | **28,493** | **4,871** | 9,720 | **35,055** |
| **No. of SI deaths** | 311 | 94 | **163** | **54** | 58 | **252** |
| **IR Mortality (95% CI)** | 0.69 (0.61-0.77) | 0.81 (0.66-0.99) | **0.57 (0.49-0.66)** | **1.10 (0.84-1.44)** | 0.59 (0.46-0.77) | **0.71 (0.63-0.81)** |
| **HR SI death unadjusted (95% CI), p value** | N/A | Ref | **0.68 (0.51-0.92), p=0.01** | **1.80 (1.36-2.38), P<0.001** | Ref | **1.17 (0.89-1.55), p=0.24** |
| **HR SI death Age, gender adjusted (95% CI), p value** | N/A | Ref | **0.72 (0.54-0.96), p=0.02** | **1.60 (1.22-2.10), p=0.001** | Ref | **0.88 (0.66-1.17), p=0.39** |
| **HR SI death fully adjusted (95% CI), p value** | N/A | Ref | **0.80 (0.60-1.07), p=0.14** | **1.68 (1.26-2.22), p<0.001** | Ref | **0.77 (0.56-1.06), p=0.12** |

The incidence rate, hazards ratio of serious infection deaths only in patients with early RA in relation to initial treatment strategies and corticosteroid use (imputed analyses). Any methotrexate strategy and No DMARD strategy were compared to any non-methotrexate strategy. Steroids compared to no steroids use.

*Fully adjusted are adjusted for age, gender, smoking status, social deprivation using (IMD), comorbidity (DM, HTN and lung disease, baseline disease severity (DAS28) and RhF/CCP seropositivity.

CCP, cyclic citrullinated peptide; DMARD: disease modifying anti-rheumatic drug; HR, hazards ratio; IMD, index of multiple deprivation; IR, incidence rate; RhF, rheumatoid factor; SI, serious infection.

**Supplementary Figure S4: SMR of serious infections related mortality (Including COVID -19)**

**Supplementary Table S7: SMR of serious infections related mortality (Including COVID -19)**

|  | NEIAA patient years | Observed SI events in the NEIAA cohort | Expected SI events | SMR | (95% CI) |
| --- | --- | --- | --- | --- | --- |
| Overall cohort | **44910.10** | **311** | **78.77** | **3.94** | **(3.53-4.39)** |
| Females: | | | | | |
| <50 | 8612.97 | 5 | 0.65 | 7.57 | (3.33-15.52) |
| 50-64 | 9890.88 | 24 | 3.66 | 6.54 | (4.40-9.40) |
| 65-79 | 8202.38 | 75 | 14.09 | 5.32 | (4.24-6.59) |
| 80+ | 1956.56 | 41 | 20.08 | 2.04 | (1.50-2.71) |
| Total females | **28662.80** | **145** | **38.50** | **3.76** | **(3.20-4.40)** |
| Males: | | | | | |
| <50 | 2874.40381 | 1 | 0.40 | 2.48 | (0.60-9.16) |
| 50-64 | 5545.139 | 19 | 3.56 | 5.33 | (3.43-7.98) |
| 65-79 | 6460.3121 | 91 | 17.12 | 5.31 | (4.33-6.45) |
| 80+ | 1367.44422 | 55 | 19.18 | 2.86 | (2.20-3.67) |
| Total males | **16247.2991** | **166** | **40.27** | **4.12** | **(3.54-4.77)** |

The standardised mortality ratio (SMR) for serious infections including COVID-19. The mortality rates are elevated in younger age groups and decrease with increasing age, though with wider confidence intervals in younger categories due to lower number of serious infection-related mortality events within this patient group.

SMR = 1 suggests no difference in mortality risk. SMR > 1 indicates a higher-than-expected mortality risk. SMR < 1 indicates a lower-than-expected mortality risk.

**Supplementary Figure S5: SMR of serious infections related mortality (Excluding COVID -19)**

**Supplementary Table S8: SMR of serious infections related mortality (Excluding COVID -19)**

|  | NEIAA patient years | Observed SI events in the NEIAA cohort | Expected SI events | SMR | (95% CI) |
| --- | --- | --- | --- | --- | --- |
| Overall cohort | **44,910.10** | **291** | **40.46** | **7.19** | **(6.41-8.04)** |
| Females: | | | | | |
| <50 | 8,612.97 | 4 | 0.34 | 11.46 | (4.65-25.13) |
| 50-64 | 9,890.88 | 22 | 1.70 | 12.90 | (8.55-18.83) |
| 65-79 | 8,202.38 | 71 | 7.25 | 9.79 | (7.76-12.19) |
| 80+ | 1,956.56 | 38 | 11.93 | 3.18 | (2.32-4.27) |
| Total females | 28,662.80 | 135 | 21.23 | 6.35 | (5.37-7.47) |
| Males: | | | | | |
| <50 | 2,874.40 | 1 | 0.20 | 4.93 | (1.19-18.20) |
| 50-64 | 5,545.13 | 17 | 1.48 | 11.46 | (7.19-17.52) |
| 65-79 | 6,460.31 | 87 | 7.43 | 11.69 | (9.48-14.28) |
| 80+ | 1,367.44 | 51 | 10.10 | 5.04 | (3.84-6.52) |
| Total males | 16,247.29 | 156 | 19.23 | 8.11 | (6.93-9.43) |

The standardised mortality ratio (SMR) for serious infections excluding COVID-19. The mortality rates show similar trends, where it is elevated in younger patients age groups and decrease with age.

SMR = 1 suggests no difference in mortality risk. SMR > 1 indicates a higher-than-expected mortality risk. SMR < 1 indicates a lower-than-expected mortality risk.

**Supplementary Table S9: Predictors of SI related deaths (secondary outcome) in fully adjusted models (imputed analyses)**

| **Predictor** | **HR** | **95% CI** | **P value** |
| --- | --- | --- | --- |
| **Age** | 1.10 | (1.08-1.11) | <0.001 |
| **Gender (female)** | 0.73 | (0.58-0.93) | 0.012 |
| **Ethnicity compared to White:** |  |  |  |
| Black | 1.35 | (0.70-2.62) | 0.36 |
| Asian | 0.67 | (0.35-1.30) | 0.24 |
| Mixed | 1.75 | (0.24-12.78) | 0.57 |
| Other | 0.42 | (0.12-1.42) | 0.16 |
| **More socially deprived (IMD quantile)** | 0.99 | (0.90-1.09) | 0.96 |
| **Smoking compared to never smoked:** |  |  |  |
| Current Smoker | 2.47 | (1.73-3.53) | <0.001 |
| Ex-Smoker | 1.68 | (1.24-2.26) | 0.001 |
| Not Known Smoking status | 1.76 | (1.11-2.80) | 0.01 |
| **Comorbidities:** |  |  |  |
| Diabetes mellitus | 1.89 | (1.47-2.43) | <0.001 |
| Hypertension | 1.28 | (1.03-1.59) | 0.02 |
| Lung disease | 2.14 | (1.65-2.77) | <0.001 |
| **Disease characteristics:** |  |  |  |
| Baseline DAS28 | 1.13 | (1.04-1.23) | <0.05 |
| RhF positive | 1.49 | (1.02-2.19) | 0.03 |
| CCP positive | 1.53 | (0.97-2.42) | 0.06 |
| Double positive (RhF and CCP) | 1.70 | (1.26-2.28) | <0.001 |

Predictors of serious infections related deaths in fully adjusted models (imputed analyses) in patients with early RA.

Presented model is adjusted for age, gender, smoking status, social deprivation using (IMD), comorbidity (DM, HTN and lung disease, baseline disease severity (DAS28) and RhF/CCP seropositivity.

CCP, cyclic citrullinated peptide; DAS28, disease activity score at baseline based on 28 joint counts; HR: hazards ratio; IMD, index of multiple deprivation; RhF, rheumatoid factor.

**Supplementary Table S10: Predictors of SI related deaths (secondary outcome) in unadjusted and age/gender adjusted models (imputed analyses)**

| **Predictor** | **Hazards ratio (HR)** | **95% CI** | **P value** | **Hazards ratio (HR)** | **95% CI** | P value |
| --- | --- | --- | --- | --- | --- | --- |
| **Un-adjusted model** | | | | **Age-Gender adjusted models** | | |
| **Age** | 1.09 | (1.08-1.11) | <0.001 | 1.09 | (1.08-1.11) | <0.001 |
| **Gender (female)** | 0.49 | (0.40-0.61) | <0.001 | 0.62 | (0.50-0.78) | <0.001 |
| **Ethnicity compared to White:** |  |  |  |  |  |  |
| Black | 0.85 | (0.49-1.47) | 0.57 | 1.62 | (0.88-3.01) | 0.12 |
| Asian | 0.29 | (0.14-0.58) | <0.001 | 0.71 | (0.36-1.40) | 0.33 |
| Mixed | 0.58 | (0.08-3.86) | 0.57 | 1.45 | (0.22-9.52) | 0.69 |
| Other | 0.22 | (0.06-0.70) | 0.01 | 0.37 | (0.11-1.21) | 0.10 |
| **More socially deprived (IMD quantile)** | 0.96 | (0.87-1.05) | 0.40 | 1.04 | (0.95-1.14) | 0.29 |
| **Smoking compared to never smoked:** |  |  |  |  |  |  |
| Current Smoker | 1.86 | (1.34-2.59) | <0.001 | 3.37 | (2.42-4.69) | <0.001 |
| Ex-Smoker | 2.40 | (1.78-3.23) | <0.001 | 1.94 | (1.44-2.63) | <0.001 |
| Not Known Smoking status | 1.81 | (1.14-2.86) | 0.01 | 1.54 | (0.95-2.49) | 0.07 |
| **Comorbidities:** |  |  |  |  |  |  |
| Diabetes mellitus | 2.90 | (2.27-3.71) | <0.001 | 1.93 | (1.50-2.47) | <0.001 |
| Hypertension | 2.66 | (2.13-3.33) | <0.001 | 1.26 | (1.00-1.59) | 0.04 |
| Lung disease | 3.86 | (3.03-4.90) | <0.001 | 2.65 | (2.07-3.39) | <0.001 |
| **Disease characteristics:** |  |  |  |  |  |  |
| Baseline DAS28 | 1.24 | (1.15-1.35) | <0.001 | 1.14 | (1.05-1.24) | <0.05 |
| RhF positive | 1.23 | (0.85-1.77) | 0.25 | 1.61 | (1.09-2.38) | 0.01 |
| CCP positive | 1.10 | (0.71-1.69) | 0.65 | 1.63 | (1.03-2.59) | 0.03 |
| Double positive (RhF and CCP) | 1.21 | (0.93-1.57) | 0.15 | 2.05 | (1.55-2.72) | <0.001 |

Predictors of serious infections related deaths in unadjusted and age/gender adjusted models (imputed analyses) in patients with early RA.

Presented model is adjusted for age, gender, smoking status, social deprivation using (IMD), comorbidity (DM, HTN and lung disease, baseline disease severity (DAS28) and RhF/CCP seropositivity.

CCP, cyclic citrullinated peptide; DAS28, disease activity score at baseline based on 28 joint counts; HR: hazards ratio; IMD, index of multiple deprivation; RhF, rheumatoid factor.
